# Anti-apoptotic proteins and catalase-dependent apoptosis resistance in nickel chloride-transformed human lung epithelial cells

**DOI:** 10.3892/ijo.2013.2004

**Published:** 2013-07-03

**Authors:** YU-XIU YANG, XIU-LING LI, LEI WANG, SHUANG-YIN HAN, YAN-RUI ZHANG, POYIL PRATHEESHKUMAR, XIN WANG, JIAN LU, YUAN-QIN YIN, LI-JUAN SUN, AMIT BUDHRAJA, ANDREW J. HITRON, SONG-ZE DING

**Affiliations:** 1 Department of Internal Medicine, Henan Provincial People’s Hospital, Zhengzhou University, Zhengzhou, Henan 450000, P.R. China; 2 Graduate Center for Toxicology, College of Medicine, University of Kentucky, Lexington, KY 40536, USA

**Keywords:** Bcl-2, Bcl-xL, apoptosis, nickel, BEAS-2B cell, oxidative stress, carcinogenesis, tumor, lung cancer

## Abstract

Chronic exposure to nickel compounds is associated with increased incidence of certain types of human cancer, including lung and nasal cancers. Despite intensive investigation, the oncogenic processes remain poorly understood. Apoptosis resistance is a key feature for tumor cells to escape physiological surveillance and acquire growth advantage over normal cells. Although NiCl_2_ exposure induces transformation of human lung epithelial cells, little information is available with regard to its molecular mechanisms, it is also not clear if the transformed cells are apoptosis resistant and tumorigenic. We explored the apoptosis resistance properties of nickel chloride-transformed human lung epithelial cells and the underlying mechanisms. The results showed that transformed BEAS-2B human lung epithelial cells are resistant to NiCl_2_-induced apoptosis. They have increased Bcl-2, Bcl-xL and catalase protein levels over the passage matched non-transformed counterparts. The mechanisms of apoptosis resistance are mitochondria-mediated and caspase-dependent. Forced overexpression of Bcl-2, Bcl-xL and catalase proteins reduced NiCl_2_-induced cell death; siRNA-mediated knockdown of their expression sensitized the cells to nickel-induced apoptosis, suggesting that Bcl-2, Bcl-xl and catalase protein expression plays a critical role in apoptosis resistance. Akt also participates in this process, as its overexpression increases Bcl-xL protein expression levels and attenuates NiCl_2_-induced apoptosis. Furthermore, transformed cells are tumorigenic in a xenograft model. Together, these results demonstrate that nickel-transformed cells are apoptosis-resistant and tumorigenic. Increased expression of Bcl-2, Bcl-xL and catalase proteins are important mechanisms contributing to transformed cell oncogenic properties.

## Introduction

Nickel is a ubiquitous environmental metal, widely used in industrial processes. Nickel compounds are important human carcinogens. Epidemiologically, chronic and professional exposure to nickel compounds is associated with increased incidence of certain human cancer, including lung and nasal cancers (1,2). Both the soluble and insoluble forms of nickel compounds are detrimental to health with the insoluble form possesses higher risk, such as nickel oxide and nickel subsulfide (3,4). In 1990, nickel is classified as established carcinogen to humans (group 1) by International Agency for Research on Cancer (IARC) (3,4).

Several mechanisms have been proposed for nickel-induced carcinogenesis. These include production of reactive oxygen species (ROS), induction of DNA damage (1,4), alteration of epigenetic changes such as histone modifications (5,6), disruption of cellular iron homeostasis (7), and activation of oncogenic pathways (8,9). Despite intensive investigation, the molecular features of nickel-induced oncogenic transformation, which are essential components in tumor initiation and progression, remain elusive.

Apoptosis (programmed cell death) is a key mechanism to maintain normal cell homeostasis, it removes cells that carry abnormal genetic information and keeps the functional integrity of organ or cell populations (10). Aberrant expression of anti-apoptotic protein is a common feature to many types of malignancy including lung, breast, prostate cancer, melanoma and leukemia. Their overexpression confers the cancer cells a growth advantage to escape from physiological surveillance and promote tumor formation (10,11). Recent studies also suggest that activation of oncogenic pathways mediate nickel chloride (NiCl_2_)-induced epithelial cell oncogenic transformation accompanied by enhanced Bcl-2, Bcl-xL protein expression (9).

Bcl-2, and Bcl-xL protein expression has been implicated in cancer and cell transformation (10,12), therefore, we hypothesized that their overexpression in transformed cells may lead to apoptosis resistance contributing to nickel-induced carcinogenesis. However, it is not clear if a metal compounds such as nickel might induce apoptosis resistance in transformed cells and the underlying molecular mechanisms, neither is it known if these cells are tumorigenic. Therefore, we investigated the role of Bcl-2, Bcl-xL and ROS scavenging enzyme in nickel-induced apoptosis resistance and the molecular mechanisms, which are necessary steps to understand nickel-induced carcinogenesis.

We demonstrate in this study that increased Bcl-2, Bcl-xL and ROS scavenging enzyme expression are important in conferring NiCl_2_-transformed BEAS-2B cell apoptosis resistance. The mechanisms are mitochondria-mediated and caspase-dependent. Transformed cells are also tumorigenic with accompanied oncogenic protein expression. The results characterize the oncogenic properties of nickel-transformed human lung epithelial cells and provide insight into nickel-induced carcinogenesis.

## Materials and methods

### Cell culture and reagents

Immortalized normal human bronchial epithelial cell line, BEAS-2B was purchased from American Type Culture Collection (ATCC, Manassas, VA). NiCl_2_-transformed BEAS-2B cells were isolated from soft agar by low dose NiCl_2_ exposure with BEAS-2B cells for 6 months (9). These cells showed anchorage-independent growth, proliferated faster, but were morphologically similar to normal BEAS-2B cells (9). BEAS-2B cells that stably express catalase (OriGene, Rockville, MD), Akt and dominant negative-Akt (DN-Akt) which is a kinase-dead (K179M) mutant, were generated by integration of catalase, wild-type Akt (Akt-C), DN-Akt expression vectors into BEAS-2B cells and selected with G418. Characterization of these cells has been described before (13,14). Tissue culture reagents were purchased from Gibco (Invitrogen, Carlsbad, CA). Cells were maintained in DMEM medium supplemented with 10% fetal bovine serum and antibiotics at 37°C in a humidified 10% CO_2_ incubator.

Nickel chloride (NiCl_2_) was purchased from Sigma Chemical Company (St. Louis, MO, USA). Polyclonal and monoclonal antibodies against phospho-Akt at Ser473 (#9275), phospho-Stat3 (#9145), Bcl-xL (#2764), catalase (#8841), HIF-1α (#3434), cleaved caspase-3 (#9661), cleaved caspase-7 (#9491), cleaved PARP (#9542), peroxiredoxin 1 (#8499) were purchased from Cell Signaling Technology (Danvers, MA). Bcl-2 was from Dako (Carpinteria, CA). SOD1 (sc-11407), SOD2 (sc-130345), β-actin (sc-47778), lamin A/C (sc-6215), Akt1 (sc-5298) and NF-κB p50 (sc-7178) were purchased from Santa Cruz Biotechnology (Santa Cruz, CA).

### Assays for apoptosis

Apoptosis was determined by Annexin V/propidium iodide (PI) staining kit (BD Pharmingen, San Jose, CA). In some experiments, due to the expression of green fluorescence protein (GFP) interfering with Annexin V-FITC reading, a double stain apoptosis detection kit using Hoechst 33342/PI staining (GenScript, Piscataway, NJ) was chosen to replace Annexin V-FITC. Briefly, cells (1×10^6^) treated with or without NiCl_2_ were collected and washed once with cold PBS, they were stained with 5 μl of Annexin V-FITC or Hoechst 33342 followed by 10 μl of PI (5 μg/ml) in binding buffer [10 mmol/l HEPES (pH 7.4), 140 mmol/l NaOH, and 2.5 mmol/l CaCl_2_] for 15 min at room temperature in dark. The apoptotic cells were determined using a Becton-Dickinson FACScan cytofluorometer. Both early apoptotic (Annexin V or Hoechst 33342-positive and PI-negative) and late apoptotic (Annexin V or Hoechst 33342-positive and PI-positive) cells were included in cell death determinations.

### Western blot analyses

Cell lysates were collected in lysis buffer (50 mM Tris-HCl, pH 7.4, 1 mM EDTA, 150 mM NaCl, 1% NP-40, 0.25% Na-deoxycholate, and 1 μg/ml of aprotinin, leupeptin and pepstatin). Proteins (20 μg) were separated on 10% SDS polyacrylamide gels. Proteins were subsequently transferred from gel onto nitrocellulose membrane (Bio-Rad, Hercules, CA). Membranes were blocked for 1 h at room temperature in Tris-buffered saline plus 0.01% Tween-20 (TBS-T) in 5% non-fat dry milk (pH 7.4). Various antibodies were diluted at 1:1,000 in TBS-T with 5% non-fat dry milk solution, incubated with membrane at 4°C overnight and washed three times with TBS-T. The secondary antibody, horseradish peroxidase (HRP)-conjugated antibodies, diluted at 1:2,500 in TBS-T with 5% non-fat dry milk were incubated with membrane at room temperature for 2–3 h. Signal was detected with an enhanced chemiluminescence detection kit (Perkin Elmer Life Sciences, Boston, MA). In each experiment, either anti-β-actin or anti-lamin A/C antibody was reprobed to monitor protein loading. Image quantification was processed by ImageJ software (NIH, Rockville, MD). In some experiments, mean densitometry values were adjusted with β-actin and expressed as fold changes over the appropriate controls.

### Quantitative RT-PCR for gene expression

Total RNA from BEAS-2B cells was extracted using TRIzol reagents as described previously (15). Reverse transcription of 2 μg of total cellular RNA was performed in a final volume of 20 μl containing 5X first strand buffer (Invitrogen), 1 mM of each dNTP, 20 units of placental RNase inhibitor, 5 μM random hexamer and 9 units of Moloney murine leukemia virus reverse transcriptase (Invitrogen). After incubation at 37°C for 45 min, the samples were heated for 5 min at 92°C to end the reaction, diluted at 1:4 and stored at −20°C until PCR. cDNA (2 μl) was subjected to real-time quantitative PCR using the real-time PCR detection systems (Bio-Rad, Hercules, CA) with SYBR-Green I (Molecular Probes, Eugene, OR) as a fluorescent reporter. Threshold cycle number of duplicate reactions was determined using PCR software and levels of selected gene mRNA expression were normalized to hypoxanthine phosphoribosyltransferase (HPRT) levels using the formula 2*^(Rt-Et)^*; where *Rt* is the mean threshold cycle for the reference gene HPRT and *Et* is the mean threshold cycle for experimental gene. Data are expressed as arbitrary units and adjusted to fold changes over the non-stimulated control cells. Primer sequences are provided in Table I.

### Measurement of mitochondrial membrane potential

Mitochondrial membrane potential (ΔΨm) was monitored using MitoProbe JC-1 assay kit (Invitrogen). 5,5′,6,6′-tetrachloro-1,1′,3,3′-tetraethylbenzimidazolyl-carbocyanine iodide (JC-1), a lipophilic cationic fluorescence dye, is capable of selectively entering mitochondria, where it forms monomers, and emits green fluorescence (FL-1) when ΔΨm is relatively low. At high ΔΨm, JC-1 aggregates and gives red fluorescence (FL-2) (16). Thus the red and green fluorescence of JC-1 reflect the change of ΔΨm of the mitochondrial membrane. Briefly, BEAS-2B or transformed BEAS-2B cells (5×10^5^) were seeded into 60-mm culture dishes and treated with NiCl_2_ for 16 h. Cells were trypsinized, washed in ice-cold PBS and incubated with 2 μM JC-1 at 37°C for 20 min. Cells were washed once with PBS and analyzed by FACScan cytofluorometer.

### Caspase activity assay

Caspase activity was assessed using the luminescent Caspase-Glo^®^ 3/7 assay system (Promega, Madison, WI) following manufacturer’s instructions. Briefly, BEAS-2B or transformed BEAS-2B cells were treated with or without NiCl_2_ at 1.5 mM for 16 h. Caspase-Glo 3/7 reagent (100 μl) was added into 96-well plates and incubated for 1 h at room temperature. The luminescence was measured using a Glomax™ 96 microplate luminometer (Promega). Z-VAD-FMK (carbobenzoxy-valyl-alanyl-aspartyl-[O-methyl]-fluoromethylketone), a cell-permeant pan-caspase inhibitor that irreversibly binds to the catalytic site of caspase proteases that inhibit induction of apoptosis was added 30 min before nickel stimulation at 20 μM solution in DMSO (Promega). Data were collected and expressed as fold change over the control without NiCl_2_ treatment.

### Small interfering RNA transfection

Transfection procedures followed manufacturer’s recommended protocol. Small interference RNA (siRNA) for Bcl-2 (sc-29214), Bcl-xL (sc-43630) were purchased from Santa Cruz Biotechnology (Santa Cruz, CA). Scrambled control and siRNA for catalase (s-2443) were obtained from Ambion (Austin, TX). Briefly, siRNA for Bcl-2, Bcl-xL, catalase and controls were incubated with Lipofectamine™ RNAiMAX in Opti-MEM I medium (Invitrogen) for 20 min at room temperature. They were then added to cell culture media without antibiotics. Media were replaced 24 h post-transfection. Cells were treated with or without NiCl_2_ for another 24 h. Cell lysates or cells were collected either for western blot or apoptosis analysis as mentioned above.

### Retrovirus infection

GFP labeled p-MIG, p-MIG-Bcl-2, pMIG-Bcl-xL retroviral vector (17) were kind gifts from Dr Stanley Korsmeyer, Dana-Farber Cancer Institute, USA (Addgene, #9044, #3541, #3544). The plasmids were transfected into 293-GPG cell using Lipofectamine 2000 reagent, viruses were produced in 293-GPG cells with subsequent centrifugation, filtration and stored at −20°C. BEAS-2B or T-BEAS-2B cells were then infected with control, Bcl-2, Bcl-xL retroviruses by adding virus solution into the cells with polybrene for 48 h before they were used for apoptosis and western blot analysis, virus infection in cells was monitored by checking GFP under fluorescence microscope.

### Tumor xenograft model

Athymic nude (nu/nu) female mice, 5 weeks old and weighing about 18 to 20 g were purchased from Jackson Laboratory (Bar Harbor, MI). Mice were handled following the University of Kentucky guidelines for care and use of laboratory animals and approved by the ethical committee. BEAS-2B or transformed BEAS-2B cells (2×10^6^) were mixed with Matrigel and injected in both side of flank region in 50 μl volume. Five animals in each group gave 10 injection sites. Mice were fed normally, the body weight, and tumor growth was monitored twice weekly until the tumor size reached 1 × 1 × 1 cm^3^ in size or 8 weeks post inoculation.

### Statistical analysis

All values are expressed as mean ± standard error (SEM). Student’s t-test was used to compare the difference between various controls and NiCl_2_-treated groups. P-value less than 0.05 was considered statistically significant.

## Results

### Transformed BEAS-2B cells have increased Bcl-2, Bcl-xL protein expression and are resistant to nickel-induced apoptosis

To evaluate Bcl-2, Bcl-xL protein expression and their role in nickel-induced apoptosis, BEAS-2B and transformed BEAS-2B (T-BEAS-2B) cells were first treated with or without 0.5, 1 and 1.5 mM of NiCl_2_ for 24 h for apoptosis induction. At 0.5, and 1 mM doses, only BEAS-2B cells showed apoptosis induction in 24 h, T-BEAS-2B cell showed little or no apoptosis (data not shown), therefore, 1.5 mM of NiCl_2_ was selected for apoptosis induction in both types of cells.

**Figure 1:**
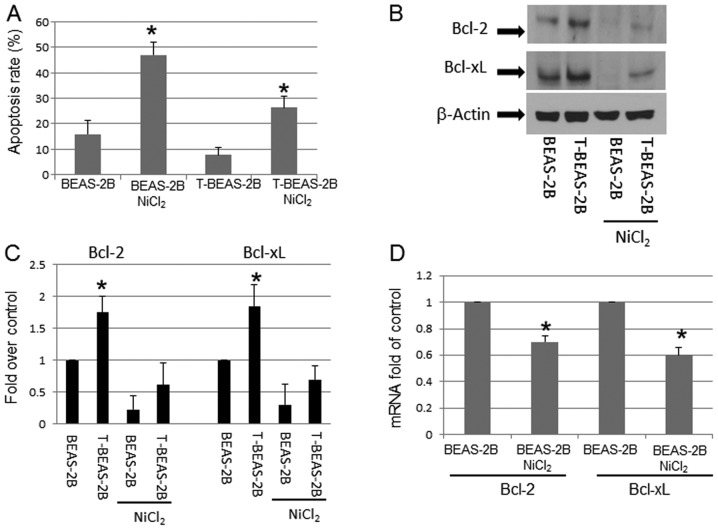
Transformed BEAS-2B cells are resistant to nickel-induced apoptosis. BEAS-2B or transformed BEAS-2B (T-BEAS-2B) cells (1×10^6^) were treated with or without 1.5 mM of NiCl_2_ for 24 h. (A) Apoptosis was analyzed from the treated cells by Annexin V/PI staining followed by flow cytometry analysis. (B) Cell lysates were collected and resolved in 10% SDS-PAGE to detect respective protein expression, arrows indicate the specific band. (C) Scanned densitometry data were expressed as arbitrary unit and adjusted as fold changes over the control. (D) Bcl-2 and Bcl-xL mRNA expression from the treated BEAS-2B cells were determined by real-time PCR as stated in Materials and methods. Data are mean ± SEM from 3–4 separate experiments. ^*^P<0.05 when compared with control cells.

BEAS-2B and T-BEAS-2B cells were treated with or without 1.5 mM of NiCl_2_ for 24 h to analysis apoptosis. The results indicated that T-BEAS-2B cells had significantly lower level of apoptosis (Fig. 1A), but increased Bcl-2, and Bcl-xL protein expression (Fig. 1B and C) when compared with control BEAS-2B cells. Following NiCl_2_ exposure, the Bcl-2, and Bcl-xL protein expression declined in both type of cells, but control BEAS-2B cells had greater level of reduction than T-BEAS-2B cells (Fig. 1B and C). To understand if Bcl-2, and Bcl-xL protein reduction might be due to NiCl_2_-induced transcriptional repression, quantitative RT-PCR assay was performed in both types of cells. Fig. 1D shows that Bcl-2 and Bcl-xL mRNA expression was repressed in BEAS-2B cells following NiCl_2_-treatment. Similar pattern of mRNA expression was also noted in T-BEAS-2B cells (data not shown). These results suggested a mechanism of NiCl_2_-induced gene transcription repression (Fig. 1D).

**Figure 2:**
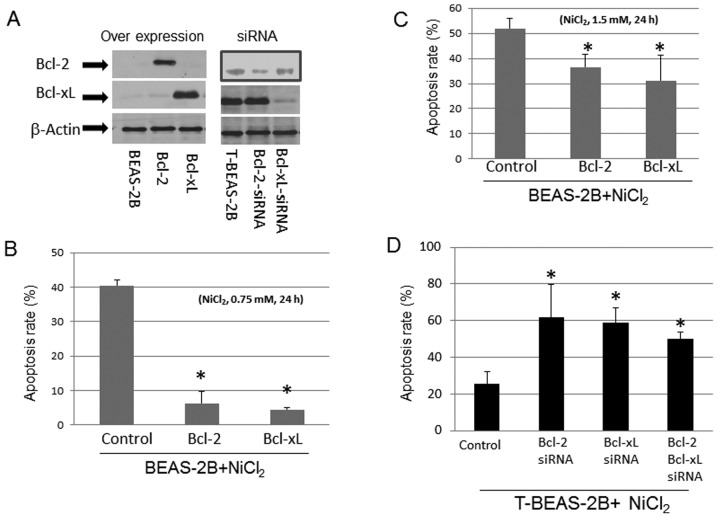
Effects of Bcl-2, and Bcl-xL expression in NiCl_2_-induced apoptosis. BEAS-2B cells were infected with control empty vector, Bcl-2, and Bcl-xL retrovirus (control, Bcl-2, Bcl-xL), transformed BEAS-2B cells (T-BEAS-2B) were transfected with control, Bcl-2, and Bcl-xL siRNA (siRNA). Cells were treated with or without NiCl_2_ at 0.75 and 1.5 mM for 24 h. To determine Bcl-2, and Bcl-xL specific protein expression, cell lysates were collected from the retrovirus infected and siRNA transfected cells (A). Protein (20 μg) was resolved in 10% SDS-PAGE to detect the respective protein expression. Blots are representative of 2–3 separate experiments with similar results, arrows indicate the specific band. Apoptosis was assayed using either Annexin V/PI (B and C) or Hoechst 33342/PI staining (D) followed by flow cytometry analysis. Data are mean ± SEM from three separate experiments, ^*^P<0.05 when compared with controls.

We tested if the levels of Bcl-2 and Bcl-xL protein expression in BEAS-2B and T-BEAS-2B cells might affect NiCl_2_-induced apoptosis. Forced overexpression of Bcl-2, and Bcl-xL protein with plasmids in BEAS-2B cells reduced NiCl_2_-induced cell apoptosis in a dose-dependent manner (Fig. 2A–C). In contrast, siRNA knockdown of Bcl-2, and Bcl-xL protein expression in T-BEAS-2B cells enhanced the apoptosis rate (Fig. 2A and D). These results suggested that the relative levels of Bcl-2, and Bcl-xL protein are important for apoptosis induction/resistance in both types of cells.

### Effects of catalase in NiCl_2_-induced apoptosis

**Figure 3:**
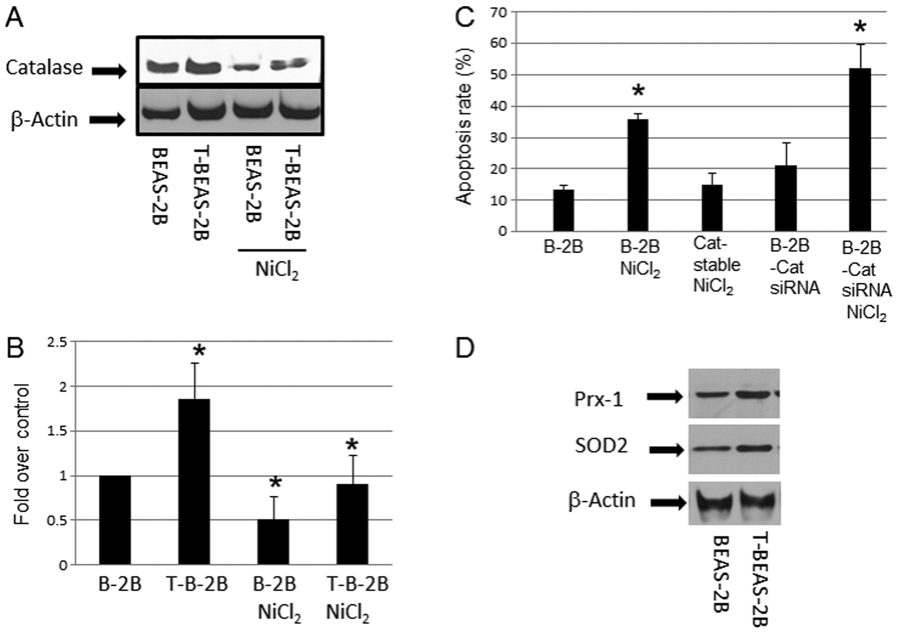
Effects of catalase in NiCl_2_-induced apoptosis. BEAS-2B (B-2B), transformed BEAS-2B (T-BEAS-2B, T-B-2B) (1×10^6^) were treated with or without NiCl_2_ at 1.5 mM for 24 h. Cell lysates were collected and resolved in 10% SDS-PAGE to detect catalase and other ROS scavenge proteins expression (A–C), blots are representative of 2–3 separate experiments with similar results. Arrows indicate the specific band. Densitometry data from catalase were scanned as arbitrary unit and adjusted as fold changes over the control (B). To determine the role of catalase in NiCl_2_-induced apoptosis (D), BEAS-2B (B-2B) cells were transfected with or without catalase siRNA (B-2B-Cat-siRNA) for 48 h. Cells were treated with or without NiCl_2_ at 1.5 mM for another 24 h. BEAS-2B catalase-stable expressing cells (Cat-stable) also received the same treatment. Apoptosis was assayed by Annexin V/PI staining followed by flow cytometry analysis (D). Data are mean ± SEM from three separate experiments, ^*^P<0.05 when compared with control cells.

After cell transformation, ROS generation is reduced in transformed cells, a mechanism that attributed to increased ROS scavenging enzyme expression (18). We examined if the apoptosis resistance in NiCl_2_-transformed cells are accompanied by higher ROS scavenging proteins expression. The results showed upregulation of catalase in nickel-transformed cells over the controls (Fig. 3A and B). Overexpression of catalase by using a stable cell line reduced NiCl_2_-induced apoptosis; in contrast, using catalase siRNA to reduce its expression (data not shown) enhanced apoptosis (Fig. 3C). These results suggested that catalase or generation of ROS is important mechanism in NiCl_2_-induced apoptosis. Western blot analysis also indicated that other ROS scavenging enzymes such as SOD2, and Prx-1 protein levels were increased in T-BEAS-2B cells when compared with control non-transformed BEAS-2B cells (Fig. 3D), while SOD1, and Phox47 protein expression showed no difference (data not shown). Thus, these results provide mechanistic explanation of reduced ROS level in T-BEAS-2B cells as reported previously (9), and these mechanisms may contribute to the apoptosis resistance in transformed cells.

### Transformed cells are resistant to NiCl_2_-induced mitochondria damage

**Figure 4:**
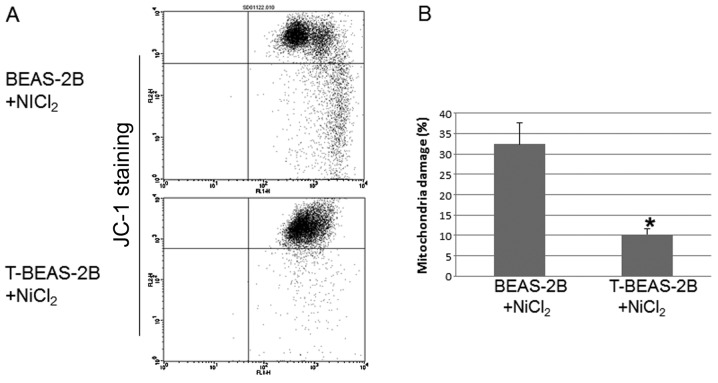

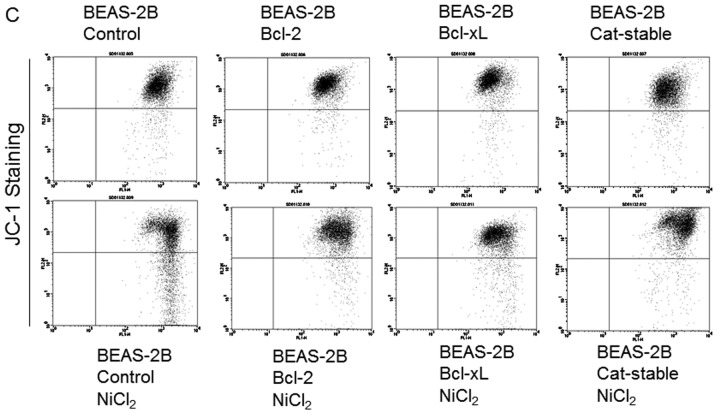

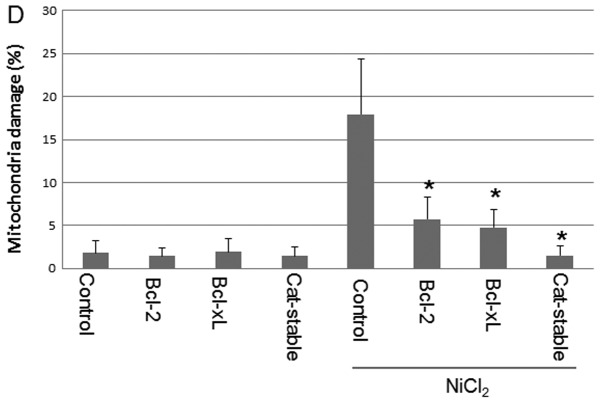
Transformed cells are resistant to NiCl_2_-induced mitochondria damage. To evaluate NiCl_2_-induced mitrochodria damage, BEAS-2B, transformed BEAS-2B (T-BEAS-2B), control empty vector, Bcl-2, and Bcl-xL-retrovirus infected BEAS-2B (control, Bcl-2, Bcl-xL) and BEAS-2B catalase-stable expressing cells (Cat-stable) (1×10^6^) were treated with or without NiCl_2_ at 1.5 mM for 16 h. Cells were stained with JC-1 dye followed by flow cytometry analysis. (A and C) Representative images are shown; (B and D) quantification of three separate experiments are expressed as mean ± SEM. (B and D) ^*^P<0.05 when compared with controls.

To investigate whether transformed cells are resistant to mitochondria damage. We evaluated NiCl_2_-induced mitochondria damage in both types of cells and the role of Bcl-2 and Bcl-xL in these processes. The results showed that T-BEAS-2B cells are more resistant to NiCl_2_-induced mitochondrial damage compared with non-transformed BEAS-2B cells (Fig. 4A and B). Forced overexpression of Bcl-2, Bcl-xL and catalase largely abolished NiCl_2_-induced mitochondria damage in BEAS-2B cells (Fig. 4C and D). Significant difference was noted when the quantitative data were compared with control cells (^*^P<0.05). These results indicated that enhanced Bcl-2, Bcl-xL and catalase protein expression are important mechanisms for reduced mitochondria damage in transformed cells.

### Caspase activation in nickel-transformed cells

**Figure 5:**
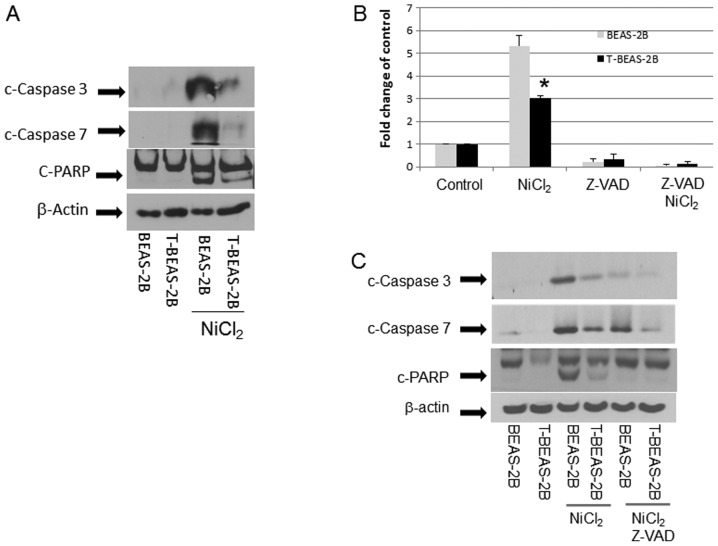

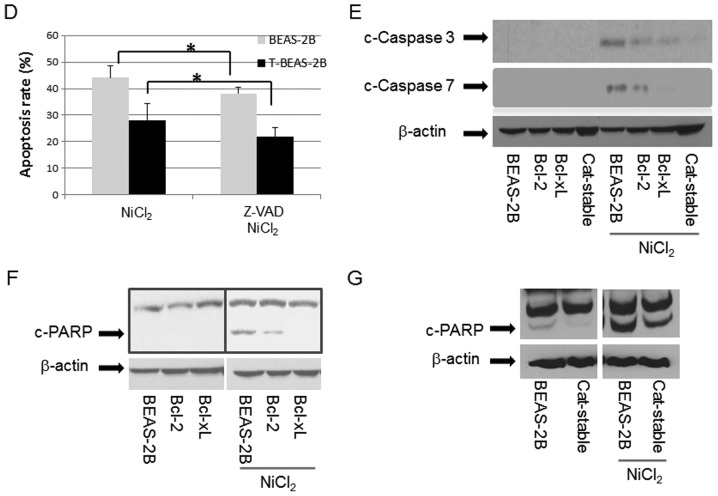
Caspase activation in nickel-transformed cells. BEAS-2B, transformed BEAS-2B (T-BEAS-2B), control empty vector, Bcl-2, and Bcl-xL-retrovirus infected BEAS-2B (Bcl-2, Bcl-xL) and BEAS-2B catalase-stable expressing cells (Cat-stable) (1×10^6^) were treated with or without NiCl_2_ at 1.5 mM for 24 h. Cell lysate was collected and 20 μg of protein resolved in 10% SDS-PAGE to detect respective protein expression (A, C, E–G). Blots are representative from 3–4 separate experiments with similar results, arrows indicate the specific band. To determine caspase 3/7 enzymatic activity, BEAS-2B and T-BEAS-2B cells were treated with or without NiCl_2_ at 1.5 mM for 16 h and enzymatic activity was assayed as described in Materials and methods (B). For apoptosis assay, BEAS-2B, transformed BEAS-2B (T-BEAS-2B) cells were treated with or without NiCl_2_ at 1.5 mM and Z-VAD for 24 h, apoptosis was assayed using the Annexin V/PI staining followed by flow cytometry (D). Quantitative data are expressed as mean ± SEM from three separate experiments, ^*^P<0.05 when compared with respective controls.

The above results demonstrated that T-BEAS-2B cells differ from BEAS-2B cells in NiCl_2_-induced apoptosis resistance and mitochondria damage; thus we further studied the underlying molecular mechanisms. We compared caspase activation in both types of cells. The result in Fig. 5 clearly indicates that upon NiCl_2_ stimulation, BEAS-2B cells had much stronger caspase activation, presented as increased c-caspase 3, 7 and c-PARP protein expression when compared with T-BEAS-2B cells (Fig. 5A). This was accompanied by increased caspase 3/7 enzymatic activity, while Z-VAD, a pan-caspase inhibitor, blocked the caspase 3/7 enzymatic activity (Fig. 5B), reduced c-caspase 3, 7 expression (Fig. 5C), and attenuated apoptosis (Fig. 5D).

In addition, we evaluated if Bcl-2, Bcl-xL and catalase proteins that are overexpressed in T-BEAS-2B cell may antagonize the NiCl_2_-induced caspase activation. The results indicated that overexpression of Bcl-2, Bcl-xL and catalase protein effectively reduced the c-caspase 3, 7 and c-PARP protein expression (Fig. 5E–G), respectively, correlating with reduced mitochondria damage and apoptosis. Therefore, these results demonstrated that upregulation of Bcl-2, Bcl-xL and catalase in T-BEAS-2B suppresses caspase activation, which are essential mechanisms underlying the adapted apoptosis resistance in these cells.

### Role of p-Akt in NiCl_2_ induced-apoptosis

**Figure 6:**
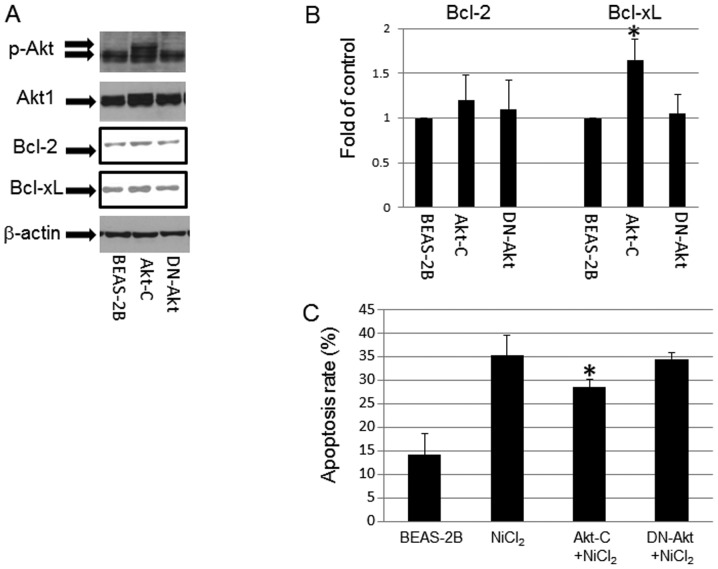
Effects of Akt expression in NiCl_2_-induced apoptosis. BEAS-2B and BEAS-2B stably expressing Akt (Akt-C), dominate negative-Akt (DN-Akt) cells were treated with or without NiCl_2_ at 1.5 mM for 24 h. Cell lysates were collected and 20 μg of protein was resolved in 10% SDS-PAGE to detect protein expression. Blots are representative of three separate experiments with similar results. (A) Arrows indicate the specific band. (B) Scanned densitometry data are expressed as arbitrary units and adjusted as fold changes over the control. (C) Apoptosis was determined by Annexin V/PI staining followed by flow cytometry analysis. Data are mean ± SEM from three separate experiments, ^*^P<0.05 when compared with control cells.

p-Akt expression was upregulated in T-BEAS-2B cells in a previous study (9). To understand its role in NiCl_2_-induced apoptosis, we used Akt overexpression (Akt-C) and dominant negative Akt (DN-Akt) stable cell lines to determine the effects of Akt on NiCl_2_-induced apoptosis. The results indicated that Akt stable cells had persistently enhanced p-Akt level (Fig. 6A), and increased Bcl-xL protein expression, but not Bcl-2 expression; while in DN-Akt cells, Bcl-2, and Bcl-xL protein expression remain at the basal control level (Fig. 6A and B). In addition, NiCl_2_ challenge induced marginally higher levels of apoptosis in control and DN-Akt cells over the Akt stable cells (Fig. 6C), pointing out the protective roles of Akt in NiCl_2_-induce BEAS-2B cell apopotsis.

### Tumorigenic properties of NiCl_2_ transformed cells

**Figure 7:**
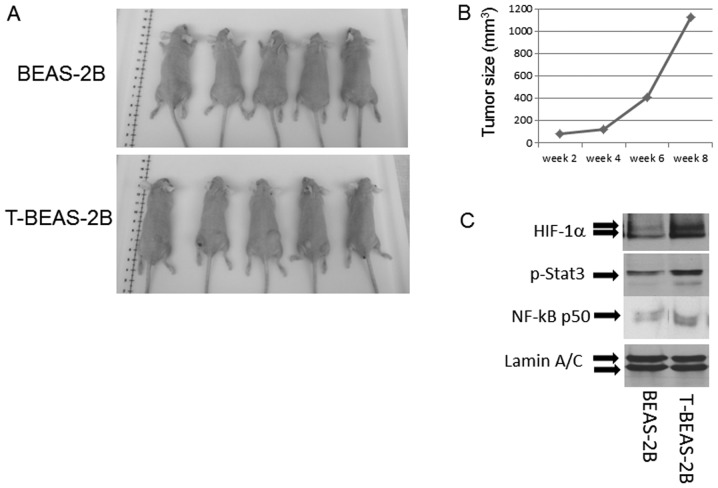
Tumorigenic properties of NiCl_2_-transformed cells. BEAS-2B or transformed BEAS-2B (T-BEAS-2B) cells (2×10^6^) were mixed with matrigel and injected subcutaneously into athymic nude mice on both sides of flank region. (A and B) Five animals were injected in each group, tumor growth were monitored twice weekly until the tumor size reach 1 × 1 × 1 cm^3^ in size or by 8 weeks post inoculation. (C) To monitor the oncogenic protein expression in transformed BEAS-2B cells, cell lysates from each type of cell were collected and resolved in 10% SDS-PAGE to detect p-Stat3, NF-κB-p50 subunit and HIF-1α protein expression. Blots are representative of 3–4 separate experiments with similar results. Arrows indicate the specific band.

To evaluate if the transformed cells are tumorigenic, we injected both control BEAS-2B and T-BEAS-2B cells into nude mice and observed the tumor growth. The results indicated that among five injected mice in each group, only T-BEAS-2B cells induced tumor growth, and tumors occurred at multiple injection sites, grew into various sizes within 8 weeks (Fig. 7A and B). Furthermore, western blot analysis revealed increased HIF-1α NF-κB-p50 subunit and p-Stat3 protein expression in T-BEAS-2B cells when compared with non-transformed cells, underlying the oncogenic features of the NiCl_2_-transformed cells (Fig. 7C).

## Discussion

Nickel is a known human carcinogen and induces genotoxic stress. A number of mechanisms have been proposed for its carcinogenic effects; such as generation of ROS, induction of DNA damage, and activation of oncogenic pathways (3). However, little information is available with regard to the mechanisms of apoptosis resistance in nickel-transformed cells. In this study, we focused on apoptosis resistant and tumorigenic property of NiCl_2_-transformed human lung epithelial cells, and explored the potential molecular mechanisms. The results demonstrate that NiCl_2_ transformed BEAS-2B cells are resistant to apoptosis and tumorigenic. Higher levels of Bcl-2, Bcl-xL and catalase protein expression are important mechanisms contributing to the transformed cell oncogenic properties.

Previously *in vitro* studies have shown that water-insoluble nickel compounds such as nickel oxide, nickel subsulfide are more readily phagocytized than the water-soluble nickel compounds such as nickel chloride. They can reach the nucleus of the cell in greater amounts than that of water-soluble nickel compounds and induce more cell damage (19,20). In the current study, NiCl_2_ was selected due to the BEAS-2B cells being transformed by NiCl_2_ exposure (9). One important characteristic of nickel compounds is the generation of ROS in host cells, and ROS appear critical in nickel-induced cell damage, such as apoptosis and oncogenic transformation (1,4). Increasing evidence has indicated that ROS plays an important role in inducing apoptosis and carcinogenesis following diverse exposure to environmental stimuli (21). However, ROS generation in transformed cells is reduced (9,18), the mechanism is not clear. In the current study, we confirm that antioxidant enzymes are increased in transformed cells including catalase, SOD2 and Prx-1 (22), suggesting that they are responsible for the reduced ROS generation in transformed cells (9,18). Higher ROS scavenging enzyme expression may also contribute to NiCl_2_-induced apoptosis resistance in transformed cells by attenuating the detrimental effects from ROS generated by NiCl_2_ exposure. However, the biological implications of these changes during transformation have yet to be explored, as well as the molecular mechanisms that result in their increased expression in transformed cells. The data provide evidence that transformation involves multiple cellular signal alternations, which deserve further investigation.

Bcl-2 and Bcl-xL proteins are overexpressed in a variety of human cancers (23), and are important members of anti-apoptotic proteins family. The mechanisms of Bcl-2 and Bcl-xL protect cells from apoptosis through either heterodimerization with pro-apoptotic proteins, or their direct pore-blocking effects on the outer membrane of mitochondria (11,23). Mitochondria play a critical role in apoptosis induction, which involves both caspase-dependent and -independent pathways. We noted that NiCl_2_-induced apoptosis can be affected by both Bcl-2 and Bcl-xL expressing vector and siRNA transfection, indicating that their relative levels regulate cell apoptosis resistance in T-BEAS-2B cells. The question of how transformed cell evolve into higher level of Bcl-2, and Bcl-xL protein expression remain to be investigated, we hypothesis that either genetic or epigenetic modifications may be responsible during the transformation and selection processes.

In our study, NiCl_2_ treatment reduced the cellular level of Bcl-2 and Bcl-xL proteins in both transformed and non-transformed cells (Fig. 1A and B), but the level of reduction in transformed cells is less dramatic, which is associated with low apoptotic rate. This effect also correlates with reduced mitochondrial stress, c-caspase 3, 7 protein level, caspase enzymatic activity and PARP cleavage. Together, they suggest that transformed cells evolve a mechanism of increased Bcl-2/Bcl-xL protein expression, reduced mitochondrial stress and alleviated caspase activation upon NiCl_2_ exposure, therefore, confer these cells the apoptosis resistant property.

Several signaling pathways such as PI3K, NF-κB, Akt, and Sonic hedgehog have been shown to regulate Bcl-2 and Bcl-xL protein expression and apoptosis in different cell types (12,24,25). Induction of cyclooxygenesis 2 is also important in nickel induced apoptosis (26). In the current study, we focus on the effects of Bcl-2, Bcl-xL, and catalase by examining their roles in apoptosis resistance in transformed cells, as they are either the effector proteins downstream of the above mentioned signal pathways or involved in ROS generation. Akt is a serine/threonine kinase, also known as protein kinase B (PKB). It is involved in regulating multiple cellular functions, including cell growth, proliferation, survival, glucose metabolism and genome stability (27,28). Aberrant Akt activation has been noted in breast, prostate, lung, pancreatic, liver, ovarian and colorectal cancers, and in malignant transformation (27–30). Akt overexpression enhances Bcl-xL expression, which is correlated with the fact that Akt-stable expressing cells are more resistant to NiCl_2_-induced apoptosis. The results uncovered anti-apoptotic protein-dependent mechanism in nickel-induced apoptosis resistance in transformed cells.

It is worth noting that overexpression of Akt could not completely block the nickel-induced apoptosis (Fig. 6), and even this effect was marginal. Since there are two major apoptotic pathways, the death-receptor pathway and the mitochondrial pathway, we consider other signal pathways might also contribute to the nickel-induced apoptosis. It has been reported that increased FasL expression, cell cycle alteration, activation of c-Myc through ERK pathway, caspase-8/AIF-mediated pathways, and activation of NF-κB/Cox2 pathway all participate in nickel-induced apoptosis (26,31–34). Therefore, each may initiate and contribute to the pathway or cell type-specific apoptosis processes in additional to the mechanisms discussed in the present study. However, increased Bcl-2, Bcl-xL and catalase protein expression, reduced mitochondria damage and caspase activation described here appear to be the essential mechanisms involved in NiCl_2_-mediated cell death resistance program in transformed BEAS-2B cells. Future investigations will be required to clarify if other mechanisms might also be involved in transformed cell resistance to apoptosis.

One important feature of transformed cells is their tumorigenic property. These cells also have enhanced p-Stat3, NF-κB-p50 subunit, and HIF-1α protein expression. The altered oncogene expression probably reflects extensive genetic or epigenetic rearrangements during the transformation processes which allow transformed cells to adopt and grow in a new environment. It is also possible that they acquire cancer stem cell trait to allow cell resistant apoptosis. Future studies focusing on nickel-induced apoptosis resistance and cell transformation are of great interest to understand nickel-induced carcinogenic mechanisms and to provide options for prevention.

## Figures and Tables

**Table I:** PCR primers.

Gene name	For RT-PCR (5′→3′)
*Bcl-2*	Fwd: GCCTTCTTTGAGTTCGGTGG
	Rev: ATCTCCCGGTTGACGCTCT
*Bcl-xL*	Fwd: GAGCTGGTGGTTGACTTTCTC
	Rev: TCCATCTCCGATTCAGTCCCT
*Hprt*	Fwd: TTGGAAAGGGTGTTTATTCCTCA
	Rew: TCCAGCAGGTCAGCAAAGAA

*Hprt*, hypoxanthine phosphoribosyltransferase.
